# Supplementary material for: Combination of Bone-Modifying Agents with Immunotarget Therapy for Hepatocellular Carcinoma with Bone Metastases
Source: J Clin Med. 2022 Nov 23;11(23):6901. doi: 10.3390/jcm11236901 (PMC9738198; doi:10.3390/jcm11236901)
Supplement: Supplementary file 1 [file jcm-11-06901-s001.zip › Supplement Figure S1.pdf]

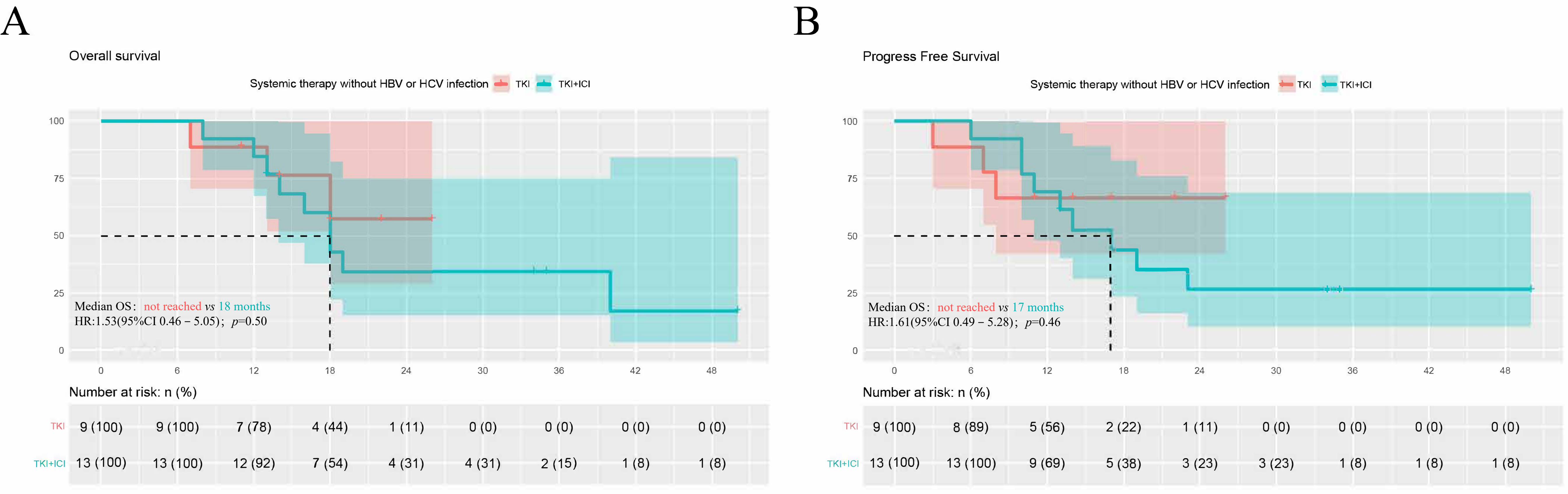
A
Overall survival
Systemic therapy without HBV or HCV infection
TKI
TKI+ICI
100
75
50
25
0
0
6
12
18
24
30
36
42
48
Median OS: not reached vs 18 months
HR:1.53(95%CI 0.46 – 5.05); p=0.50
Number at risk: n (%)
TKI 9 (100) 9 (100) 7 (78) 4 (44) 1 (11) 0 (0) 0 (0) 0 (0) 0 (0)
TKI+ICI 13 (100) 13 (100) 12 (92) 7 (54) 4 (31) 4 (31) 2 (15) 1 (8) 1 (8)
B
Progress Free Survival
Systemic therapy without HBV or HCV infection
TKI
TKI+ICI
100
75
50
25
0
0
6
12
18
24
30
36
42
48
Median OS: not reached vs 17 months
HR:1.61(95%CI 0.49 – 5.28); p=0.46
Number at risk: n (%)
TKI 9 (100) 8 (89) 5 (56) 2 (22) 1 (11) 0 (0) 0 (0) 0 (0) 0 (0)
TKI+ICI 13 (100) 13 (100) 9 (69) 5 (38) 3 (23) 3 (23) 1 (8) 1 (8) 1 (8)
