# Supplementary material for: Combination of Bone-Modifying Agents with Immunotarget Therapy for Hepatocellular Carcinoma with Bone Metastases
Source: J Clin Med. 2022 Nov 23;11(23):6901. doi: 10.3390/jcm11236901 (PMC9738198; doi:10.3390/jcm11236901)
Supplement: Supplementary file 1 [file jcm-11-06901-s001.zip › Supplement Figure S2.pdf]

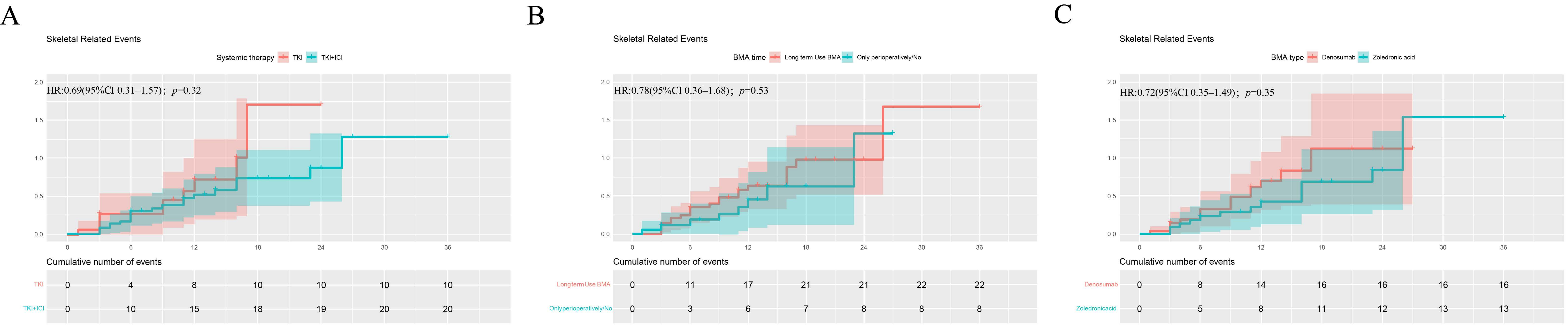
A
Skeletal Related Events
Systemic therapy TKI TKI+ICI
HR:0.69(95%CI 0.31–1.57); p=0.32
2.0
1.5
1.0
0.5
0.0
0 6 12 18 24 30 36
Cumulative number of events
TKI 0 4 8 10 10 10 10
TKI+ICI 0 10 15 18 19 20 20
B
Skeletal Related Events
BMA time Long term Use BMA Only perioperatively/No
HR:0.78(95%CI 0.36–1.68); p=0.53
2.0
1.5
1.0
0.5
0.0
0 6 12 18 24 30 36
Cumulative number of events
Long term Use BMA 0 11 17 21 21 22 22
Onlyperioperatively/No 0 3 6 7 8 8 8
C
Skeletal Related Events
BMA type Denosumab Zoledronic acid
HR:0.72(95%CI 0.35–1.49); p=0.35
2.0
1.5
1.0
0.5
0.0
0 6 12 18 24 30 36
Cumulative number of events
Denosumab 0 8 14 16 16 16 16
Zoledronicacid 0 5 8 11 12 13 13
